# Comprehensive analysis of lactylation-related gene sets and mitochondrial functions in gastric adenocarcinoma: implications for prognosis and therapeutic strategies

**DOI:** 10.3389/fimmu.2024.1451725

**Published:** 2024-10-16

**Authors:** Xindong Yin, Wenya Xing, Nan Yi, Yuanzi Zhou, Yue Chen, Zhiwei Jiang, Chaoqun Ma, Cunbing Xia

**Affiliations:** Department of General Surgery, Affiliated Hospital of Nanjing University of Chinese Medicine, Jiangsu Province Hospital of Chinese Medicine, Nanjing, China

**Keywords:** gastric adenocarcinoma, lactylation, mitochondrial dysfunction, tumor microenvironment, prognostic biomarkers

## Abstract

Gastric adenocarcinoma (STAD) is characterized by high heterogeneity and aggressiveness, leading to poor prognostic outcomes worldwide. This study explored the prognostic significance of lactylation-related gene sets and mitochondrial functions in STAD by integrating large-scale genomic datasets, including TCGA and several GEO datasets. We utilized Spatial transcriptomics and single-cell RNA sequencing to delineate the tumor microenvironment and assess the heterogeneity of cellular responses within the tumor. Additionally, the study identified distinct molecular subtypes within STAD that correspond with unique survival outcomes and immune profiles, enhancing the molecular classification beyond current paradigms. Prognostic models incorporating these molecular markers demonstrated superior predictive capabilities over existing models across multiple validation datasets. Furthermore, our analysis of immune landscapes revealed that variations in lactylation could influence immune cell infiltration and responsiveness, pointing towards novel avenues for tailored immunotherapy approaches. These comprehensive insights provide a foundation for targeted therapeutic strategies and underscore the potential of metabolic and immune modulation in improving STAD treatment outcomes.

## 1 Introduction

Gastric cancer (GC), particularly gastric adenocarcinoma (STAD), stands as a significant public health challenge worldwide (1, 2). It is the fifth most common cancer and the third most common cause of cancer-related deaths globally (3, 4). The aggressive nature of the tumor and the disease’s often late diagnosis strongly contribute to its mortality rate (5). Patients with advanced STAD continue to have a poor prognosis, with a poor five-year survival rate, despite improvements in surgical methods and systemic therapies (6–8). While our primary focus is on gastric adenocarcinoma (STAD), we also included GEO datasets from lung adenocarcinoma and urothelial carcinoma to explore whether the observed lactylation-related gene expressions and their impact on immune response were consistent across different cancer types. This comparative analysis aims to provide a broader understanding of lactylation’s role in cancer biology. Among the lactylation-related genes identified, PTMA was selected for further experimental validation due to its significant association with mitochondrial dysfunctions and its prognostic value in gastric adenocarcinoma. PTMA’s role in immune modulation and cancer progression makes it a promising candidate for understanding the molecular mechanisms underlying lactylation’s impact on cancer biology.

The complexity of gastric cancer, characterized by its genetic, epigenetic, and environmental heterogeneity, complicates effective treatment strategies (9, 10). The integration of molecular biology and gene expression profiling has started to illuminate the diverse molecular mechanisms underlying the pathogenesis of STAD (11, 12). These insights have led to the classification of gastric cancer into distinct molecular subtypes, each with unique prognostic and therapeutic implications (13–15). However, the clinical application of these classifications and the development of targeted therapies have been hindered by a limited understanding of the molecular drivers and systemic immune responses’ interaction with the tumor microenvironment.

Recent advancements in high-throughput technologies and bioinformatics tools have provided unprecedented opportunities to explore the complex biological landscape of gastric cancer (16, 17). Transcriptomic profiling, particularly through Gene Expression Omnibus (GEO) datasets and the Cancer Genome Atlas (TCGA), has offered valuable resources for identifying key molecular signatures and pathways that could serve as potential diagnostic, prognostic, and therapeutic targets (18, 19).

One of the pivotal aspects of this research is the study of gene expression modulation via post-translational modifications (PTMs), such as lactylation, which have recently been recognized for their roles in cancer biology (20, 21). Lactylation, a relatively new addition to the list of PTMs, has been implicated in various cellular processes, including metabolism, immune response, and gene expression regulation (22, 23). Exploring lactylation-related gene sets in STAD may help identify novel aspects of gastric cancer pathophysiology and identify promising targets for therapy.

Furthermore, the tumor microenvironment (TME), which includes a complex array of fibroblasts, immune cells, and other stromal elements, plays a vital role in the progression and response to therapy in gastric cancer (24, 25). Modern methods like spatial transcriptomics and single-cell RNA sequencing (scRNA-seq) provide a thorough analysis of the tumor microenvironment (TME), offering valuable information about the cellular variability and dynamic interactions inside the tumor that contribute to cancer progression and treatment resistance (26–28).

In this context, the prognostic value of gene expression profiles has been increasingly recognized. Building robust prognostic models based on differential gene expression could significantly improve the stratification of patients for tailored therapeutic strategies. Additionally, with the advent of immunotherapy as a powerful modality in cancer treatment, understanding the interaction between the immune landscape of STAD and its molecular subtypes could guide the development of more effective immune-based therapies.

However, despite these technological advancements, the translation of molecular findings into clinical practice remains slow, and the impact on patient survival has been modest. This underscores the need for continued research into the molecular mechanisms of gastric cancer, leveraging the latest technologies to bridge the gap between bench research and bedside application.

The present study aims to address these challenges by employing comprehensive bioinformatic analyses to explore the correlations between lactylation-related gene expressions and mitochondrial-related genes and to identify those with prognostic significance in gastric cancer. By integrating data from TCGA and multiple GEO datasets, we utilized the TCGA dataset as the primary training set for constructing the prognostic model. The GEO datasets were subsequently used for validation to assess the robustness and generalizability of our findings. This research seeks to refine the molecular classification of gastric cancer, enhance the understanding of its biological underpinnings, and identify novel prognostic markers and therapeutic targets. By applying cutting-edge technologies such as scRNA-seq and spatial transcriptomics, the study will dissect the complex interactions within the gastric cancer microenvironment, offering new perspectives on the cellular processes that govern tumor behavior and response to treatment. Lactylation, a newly recognized post-translational modification, has been implicated in the regulation of various metabolic processes crucial for cancer cell survival and proliferation. In the context of cancer, particularly gastric adenocarcinoma (STAD), lactylation can significantly impact metabolic pathways, including glycolysis, oxidative phosphorylation (OXPHOS), and the tricarboxylic acid (TCA) cycle. This modification can promote the glycolytic phenotype of cancer cells, known as the Warburg effect, which supports rapid cell growth and proliferation by enhancing glucose uptake and lactate production. Additionally, lactylation has been associated with alterations in mitochondrial function, leading to a shift from oxidative phosphorylation to glycolysis, thereby influencing the overall metabolic reprogramming in cancer cells. Understanding the role of lactylation in these metabolic pathways could reveal novel insights into cancer metabolism and potential therapeutic targets.

## 2 Materials and methods

### 2.1 Acquisition and processing of transcriptomic data

RNA expression profiles and corresponding clinical data for gastric adenocarcinoma (STAD) were selected from the TCGA database, comprising 350 samples as the training set. This set was utilized for model construction, while its stability and accuracy were assessed in a validation group. All data were log2-transformed after being converted to TPM format for further analysis. Additionally, chip datasets from the GEO database were used for validation, including GSE15459 (n=192), GSE15460 (n=248), GSE57303 (n=70), GSE62254 (n=300), and GSE84437 (n=433), with GSE55696 (T=56, N=19) and GSE79973 (T=10, N=10) specifically for differential gene analysis. The normalizeBetweenArrays function from the limma package was utilized to standardize the data across chip datasets. In addition to the TCGA-STAD dataset, we analyzed GEO datasets from lung adenocarcinoma (GSE91061, GSE78220) and urothelial carcinoma (IMvigor210) to examine the potential impact of lactylation-related gene expressions on immune response across various cancer types. This was intended to validate our findings in STAD and investigate whether similar patterns could be observed in other cancers.

### 2.2 Acquisition and processing of single cell and spatial transcriptomics data

Single-cell datasets were sourced from the GEO database under GSE184198, encompassing one primary tumor sample with 13,424 cells. R software and R packages, including Seurat, were used to analyze the data. Quality control criteria for cells included mitochondrial content under 20% and limits for UMI counts, and gene counts set between 200-30,000 and 200-5,000, respectively. Data normalization, selection of 2,000 variable genes, and scaling were conducted using Seurat’s NormalizeData, FindVariableFeatures, and ScaleData functions, with cell cycle effects regressed out (vars.to.regress = c(“S.Score”, “G2M.Score”)). The subsequent analysis involved dimension reduction techniques UMAP and t-SNE, and the Louvain clustering algorithm, all implemented via Seurat. To find differential genes between cell types or clusters, the FindAllMarkers function was used, with thresholds set at log2FC > 0.25, expression proportion > 0.1, and p-value < 0.05. Spatial transcriptomics data were obtained from GEO’s GSE251950, comprising 10 tumor samples analyzed using quality-controlled results from SpaceRanger software. Data transformation, normalization, and highly variable gene selection were performed using the SCTtransform algorithm, with average spot numbers at 3229 and average UMI, gene counts, and mitochondrial content at 9885.8, 3372.8, and 2%, respectively. Analysis and visualization were conducted using Seurat software. The conditional autoregression-based deconvolution (CARD) algorithm was used for deconvolution analysis, utilizing single-cell annotation data to predict cell types for each spot in spatial data. Visualization of cell types in spatial datasets was performed using CARD software. The AUCell package was employed to calculate the activity scores for gene signatures related to lactylation, immune response, and stromal characteristics. These scores were used to evaluate the enrichment of these signatures across different cell types, providing insights into their functional implications within the tumor microenvironment.

### 2.3 Cell annotation analysis

Initially, we identified markers for various cell types: epithelial cells (“EPCAM,” “KRT18”, “KRT19”, “CDH1”); fibroblasts (“DCN,” “THY1”, “COL1A1”, “COL1A2”); endothelial cells (“PECAM1”, “CLDN5”, “FLT1”, “RAMP2”); T cells (“CD3D”, “CD3E”, “CD3G”, “TRAC”); NK cells (“NKG7”, “GNLY,” “NCAM1”, “KLRD1”); B cells (“CD79A”, “IGHM,” “IGHG3”, “IGHA2”); and mast cells (“KIT”, “MS4A2”, “GATA2”). We specifically isolated and clustered epithelial cells based on these markers to investigate tumour heterogeneity. To illustrate these analyses, various visualizations were created, including UMAP, t-SNE, bar charts, and heatmaps.

### 2.4 Acquisition of lactylation gene sets and mitochondrial pathways

We acquired 332 lactylation-related genes from the “MSigDB database”. Additionally, we retrieved a set of 177 human mitochondrial-related genes from the msigdbr package and utilized the ssGSEA algorithm to calculate their scores. After filtering out gene sets containing fewer than five genes, 170 gene sets remained for further analysis.

### 2.5 Prognostic gene identification and consensus clustering analysis

We performed a correlation analysis between the scores of 332 lactylation-related genes and 170 mitochondrial-related gene sets, identifying 304 genes associated with mitochondria. Subsequent univariate Cox analysis was conducted with TCGA and five GEO validation datasets, from which 12 genes were identified as having prognostic significance (p < 0.05 in at least three datasets). Clustering analysis using these 12 prognostic genes was performed in the TCGA-STAD cohort using a method called nonnegative matrix factorization (NMF), executed by the NMF package. The optimal number of clusters was determined using the cophenetic correlation. Based on the bioinformatic analysis, PTMA was selected for wet lab experiments because it was identified as one of the 12 prognostic genes showing significant differential expression and association with mitochondrial dysfunctions. Its involvement in immune regulation in gastric cancer was further explored through *in vitro* experiments to validate its potential role in cancer progression and therapeutic targeting.

### 2.6 SNV analysis

Single nucleotide variant (SNV) mutation data were downloaded from the TCGA database. To compare samples’ tumor mutation burdens (TMB), the maftools package was utilized. Furthermore, we used the Wilcoxon test to do a differential analysis between the risk groups, setting the significance level at p < 0.05.

### 2.7 Analysis of cell communication

The CellChat package was utilized to evaluate communication between cells. To generate a CellChat object, the CellChat function was used to import the normalized gene expression matrix. ProjectData, identifyOverExpressedGenes, and identifyOverExpressedInteraction functions were used to preprocess the data using their default settings. Subsequently, potential ligand-receptor interactions were identified using computeCommunProb, filterCommunication, and computeCommunProbPathway functions. Finally, the aggregateNet function was used to generate cell communication networks.

### 2.8 Differential gene analysis and enrichment analysis

Differential gene expression between tumor and adjacent normal samples in the GEO and TCGA datasets was computed using the limma package. A gene was considered significant if its absolute fold change was more than 1.2 and its adjusted p-value (Padj) was less than 0.05. Enrichment analysis for upregulated and downregulated genes was performed separately using the clusterProfiler package, employing the GSEA algorithm. Functional databases included HALLMARK, GOBP, and KEGG, with functional signatures sourced from the msigdb database. The enrichplot package was used to visualize the enrichment results.

### 2.9 Establishment of tumor-related risk features

A total of 101 different machine learning algorithm combinations were evaluated to create a prognostic model. The final model was selected based on the highest average C-index across the testing sets, enabling the risk score to be created for each patient. The prognostic model was initially constructed using the TCGA dataset, which included 350 samples. This model was then validated using five independent GEO datasets (GSE15459, GSE15460, GSE57303, GSE62254, and GSE84437). The same model parameters and thresholds were consistently applied across all datasets to ensure comparability and validity of the results. The TCGA and other cohorts’ cutoff values for grouping patients into high-risk and low-risk groups were determined using the surv_cutpoint function. We then studied how predictions between the two groups varied and assessed the model’s accuracy.

### 2.10 Risk features generated by machine learning-based ensemble methods

We developed the highly accurate and stable AI-Driven Prognostic Signature (AIDPS) model using 10 machine learning algorithms and 101 algorithm combinations. The combined algorithms included Supervised Principal Component (SuperPC), Generalized Boosting Regression Model (GBM), Cox Partial Least Squares Regression (plsRcox), CoxBoost, Ridge, Lasso, Stepwise Cox, Random Survival Forest (RSF), Elastic Net (Enet), and Survival Support Vector Machine (survival-SVM). The signature was generated as follows: Univariate Cox regression analysis was conducted to (a) identify prognostic genes across six datasets, including TCGA-STAD (as previously mentioned); (b) fit predictive models in the TCGA-STAD cohort using 101 algorithm combinations within a leave-one-out cross-validation (LOOCV) framework; (c) test each model across five validation datasets (GSE datasets); and (d) calculate Harrell’s Concordance Index (C-index) for each model across all validation datasets, selecting the model with the highest average C-index as the optimal one.

### 2.11 Prediction of immune therapy response and IPS analysis and immune checkpoint analysis

The prediction of immune therapy responses involved gathering datasets from GSE91061 (lung adenocarcinoma), GSE78220 (lung adenocarcinoma), IMvigor210 (urothelial carcinoma, UC), and Braun (renal cell carcinoma, RCC), and calculating risk scores within each dataset to predict immune therapy responses. Additionally, immune responses in the TCGA dataset were predicted using the TIDE online analysis tool (http://tide.dfci.harvard.edu/). Relevant Immune Prediction Score (IPS) data were obtained from the TCIA database to examine differences in IPS across risk groups. Correlations were analyzed between the expression levels of immune checkpoint genes “HAVCR1”, “CD28”, “ICOS,” “TNFRSF9”, “IL2RB”, “CD27”, “TNFSF14”, “CD40”, “TNFSF18”, “TNFRSF18”, “CD276”, “PVR,” “VTCN1”, “CD200”, “C10orf54”, “CD200R1”, “BTLA,” “IDO1”, “TIGIT,” “LAG3”, “CD80”, “CD86”, “LAIR1”, “ADORA2A”, “CTLA4”, “KIR3DL1”, “CEACAM1”, and risk scores.

### 2.12 Tumor immune infiltration analysis

The IOBR package was used to assess the level of immune infiltration in STAD patients using data from six evaluation methods (CIBERSORT, TIMER, MCPcounter, Estimate) and the TCGA database. Heatmaps were created with this data to measure the relative amounts of immune cell infiltration into the tumor microenvironment (TME). The Estimate algorithm’s output allowed for comparing the relative abundances of tumor, immune, and stromal cells across various risk categories.

### 2.13 Drug sensitivity analysis

The R package “oncoPredict” enabled investigators to evaluate the association between risk ratings and dose sensitivity by calculating a popular chemotherapeutic drug’s half-maximal inhibitory concentration (IC50). The Wilcoxon rank-sum test was used to compare the IC50 values between the two risk groups.

### 2.14 Patients and specimens

Tissue samples from STAD patients were systematically collected at Jiangsu Province Hospital of Chinese Medicine (Nanjing, China). Patients who underwent surgery as the primary mode of treatment and who had completed clinical and follow-up data met the inclusion criteria. Patients who had already received preoperative chemotherapy or who had additional malignant tumors were excluded. A total of 30 patients were selected for the database. Both cancerous and paracancerous tissues resected during the operation were collected for the study. The study protocol was approved by the Jiangsu Province Hospital of Chinese Medicine’s Ethics Committee (approval no. 2022NL12902), and informed consent was obtained from each participating patient.

### 2.15 Cell culture and transfection

**Figure 1:**
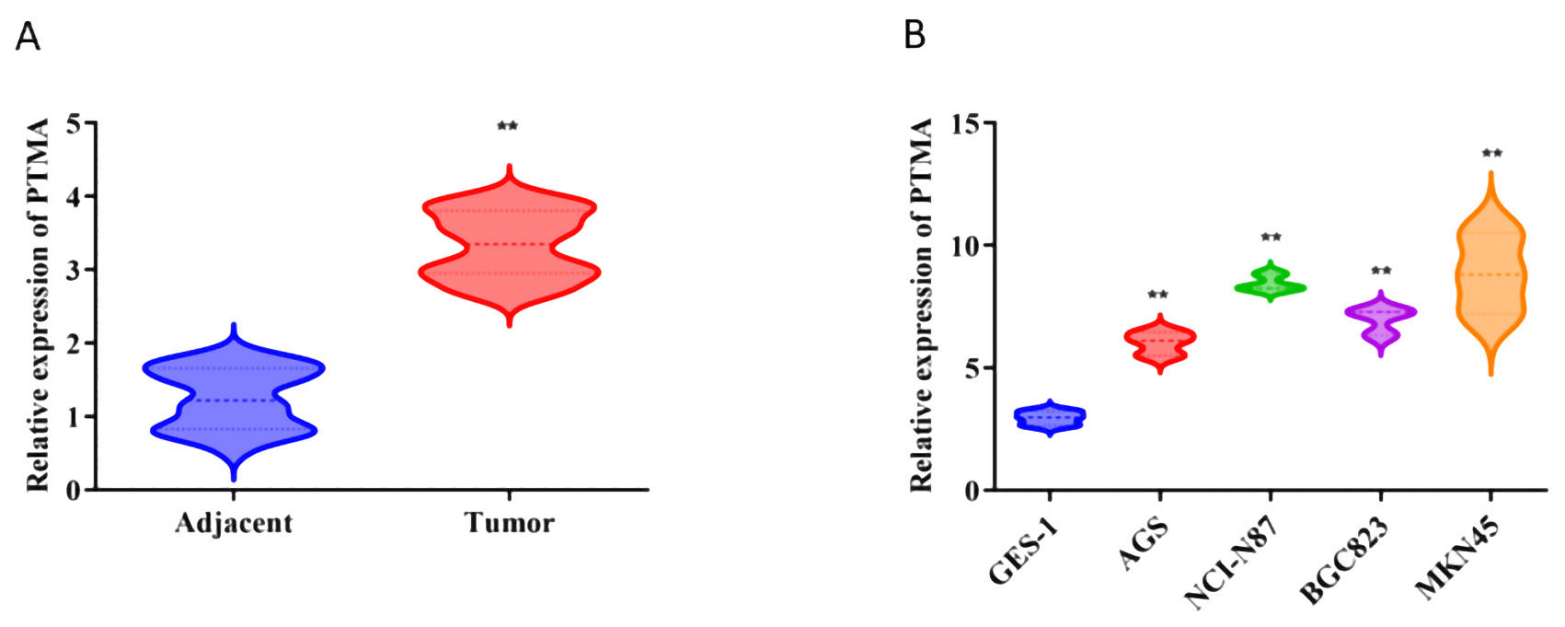
**(A)** Relative expression of PTMA in adjacent normal tissues and tumor tissues: The comparison was made between adjacent normal tissues (blue) and tumor tissues (red). Statistical significance: “**” indicates *p* < 0.01 between adjacent and tumor groups. **(B)** Relative expression of PTMA in different cell lines: The comparison was made between the normal gastric mucosa cell line GES-1 (blue) and gastric cancer cell lines AGS (red), NCI-N87 (green), BGC-823 (purple), and MKN45 (orange). Statistical significance: “**” indicates *p* < 0.01 compared to GES-1. “**” denotes statistical significance (“*” *p* < 0.05, “**” *p* < 0.01). Sample sizes are indicated within the plots. Statistical comparisons were made using the Student’s t-test.

GES-1 and BGC-823 cells were sourced from the Chinese Academy of Sciences Cell Bank, while AGS, NCI-N87, and MKN45 cells were obtained from Procell Life Science & Technology (Wuhan, China). The GES-1, MKN45, and NCI-N87 cells were kept in the RPMI-1640 culture medium (Procell Life Science & Technology, China), whereas BGC-823 and AGS cells were cultured in a DMEM high glucose medium. Incubated at 37°C in a 5% CO2 environment, all cell lines were supplemented with 10% fetal bovine serum (Procell Life Science & Technology, China). Subsequently, 1 µg of short hairpin (sh)RNA targeting PTMA (sh-PTMA; Guangzhou RiboBio Co., Ltd.) and 1 µg of negative control shRNA (sh-NC; Guangzhou RiboBio Co., Ltd.) were transfected into MKN45 and NCI-N87 cells. Real-time quantitative PCR was used to verify the transfection efficiency (**Figure 1A**).

### 2.16 Real-time PCR

Trizol reagent (TaKaRa Bio Inc., Japan) was used to extract total RNA from tissues or cells, and a two-step RNA reverse transcription kit (TaKaRa Bio Inc., Japan) was utilized for transforming the extracted RNA into cDNA. The cDNA and primers were mixed with RT-PCR SYBR Green (TaKaRa Bio Inc., Japan) for the RT-PCR reaction. The reaction was conducted with the following cycling parameters: an initial denaturation at 95°C for 30 seconds, followed by 40 cycles of denaturation at 95°C for 5 seconds, annealing at 60°C for 30 seconds, and extension at 72°C for 30 seconds.

### 2.17 Cell proliferation assay

With the use of the CCK-8 kit (Seven, China), cell viability was evaluated. A 96-well plate was seeded with a single-cell suspension at a density of 5 × 10³ cells per well. After that, each well was filled with a volume of 10 μL of CCK-8 solution every 24 hours, and each well was left to incubate for two hours. A multifunctional enzyme-linked immunosorbent assay reader was used to detect the optical density (OD) at 450 nm.

### 2.18 Cell apoptosis

Following the manufacturer’s instructions, a Cell Apoptosis Detection Kit with Annexin V-mCherry and SYTOX Green (Beyotime, Shanghai, China) was used to identify cell apoptosis. After incubating with Annexin V-mCherry and SYTOX Green for 20 minutes in a light-proof conditions, cells were rinsed with PBS and combined with 400 µL of binding buffer for 30 minutes. An FACSCanto II flow cytometer (BD Biosciences, San Jose, CA) was used to analyze the apoptosis rate.

### 2.19 Cell migration and invasion assays

Transwell chambers having a pore size of 8.0 μm were utilized to measure cell invasion and migration (Procell Life Science & Technology, China). A 500 μL medium containing 10% FBS was added to the lower chamber, while 1 × 10^4^ cells were seeded onto the upper chamber in a serum-free medium. For invasion assays, the transwell membrane was coated with 1 mg/ml Matrigel (Procell Life Science & Technology, China). After a 24-hour incubation at 37°C, cotton swabs were used to delicately remove non-migrating or non-invading cells. Crystal violet was utilized to stain and count the cells that invaded or migrated to the bottom of the membrane. The cells were preserved with 4% paraformaldehyde.

### 2.20 Wound healing assay

A 6-well plate was seeded with cells, which were then cultured until they reached 100% confluence. A scratch was produced in the cell monolayer using a pipette tip. The cells were cultivated in a serum-free medium for 24 hours following PBS washing. Images were captured at 0 and 48 hours, and cell mobility within the scratched area was analyzed using Image J.

### 2.21 Western blot

For 30 minutes, cell lysates were produced on ice using the radioimmunoprecipitation assay (RIPA) buffer. Then, using a bicinchoninic acid (BCA) kit (Beyotime, China), protein concentrations were calculatedSample preparation involved mixing the protein solution with 5× loading buffer (Beyotime, China) at a 1:4 ratio and heating the mixture for 10 minutes at 95°C. The proteins were first separated on a 10% SDS-PAGE gel and transferred to PVDF membranes (Millipore, USA). The membranes were blocked with 5% skim milk for two hours at room temperature. Following blocking, the membranes were incubated with the following primary antibodies: β-actin (1:5000; Proteintech, USA), c-caspase3 (1:1000; Abcam, USA), Bax (1:1000; Proteintech, USA), Bcl-2 (1:1000; Abcam, USA), E-cadherin (1:1000; PTM Biolabs, China), and Vimentin (1:1000; PTM Biolabs, China) at 4°C for 12 hours. The membranes were treated with primary antibodies for one hour, followed by two hours of washing and secondary antibody incubation at room temperature. Enhanced chemiluminescence (Thermo Scientific, USA) was used to visualize protein bands.

### 2.22 Statistical analysis

All data processing, statistical analysis, and graphing were done using R software version 4.1.3. Pearson correlation coefficients were used to assess the correlation between two continuous variables. The T-test or the Wilcoxon rank-sum test was used to compare continuous variables, while the chi-square test was used to analyze categorical variables. We utilized the survival package to do Kaplan-Meier and Cox regression analyses.

## 3 Results

### 3.1 Characterization of target gene sets

**Figure 2:**
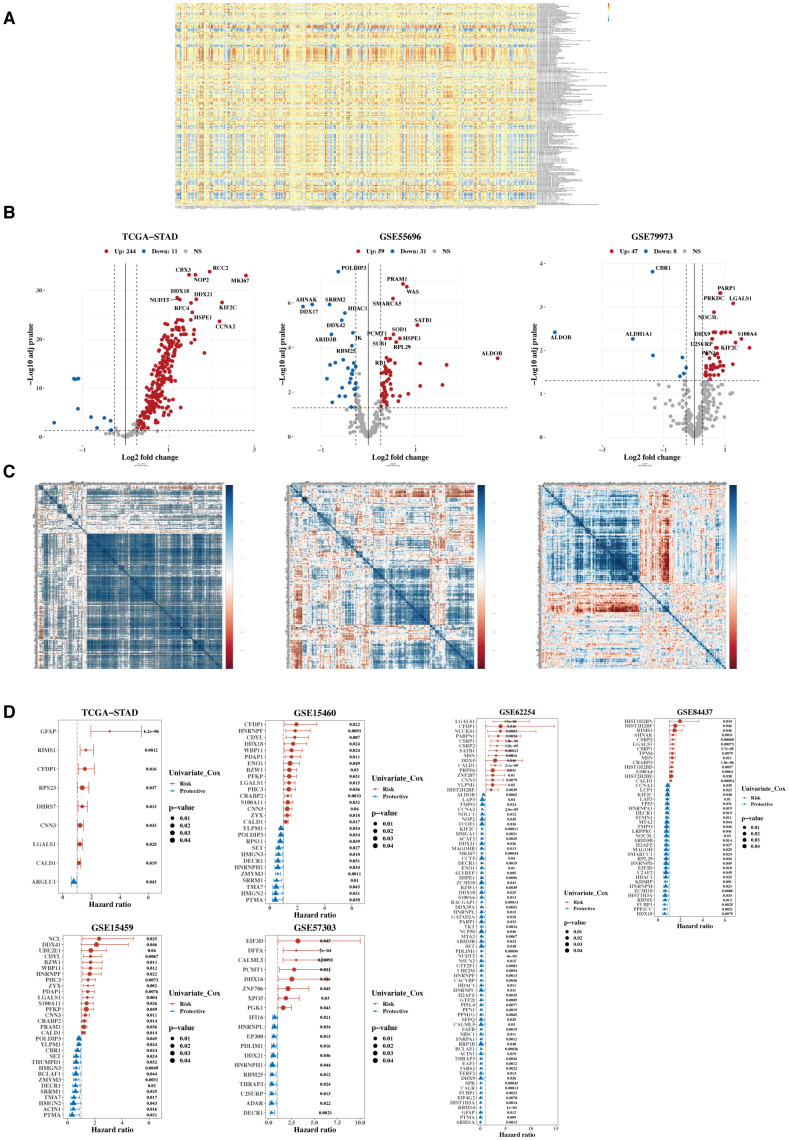
Characterization of target gene sets. **(A)** Correlation heatmap between 332 lactylation genes and 170 mitochondrial-related gene sets. **(B)** Volcano plots were generated to illustrate the differential expression of lactylation genes between tumors and adjacent normal tissues in the TCGA, GSE55696, and GSE79973 datasets. **(C)** Heatmap of expression correlation of differential genes across TCGA, GSE55696, and GSE79973. **(D)** Forest plot of hazard ratios (HR) for the combined analysis in TCGA and five GEO validation datasets.

The heatmap illustrates the correlation between 332 lactylation-related genes and 170 mitochondrial-related gene sets (**Figure 2A**). A total of 304 lactylation-related genes were identified as relevant, which associated with tumor microenvironment regulation, immune response modulation, cell proliferation and apoptosis, metabolic reprogramming, and potential prognostic markers for patient outcomes. Subsequently, their expression differences between tumors and adjacent normal tissues were analyzed using data from TCGA, GSE55696, and GSE79973 (**Figure 2B**), identifying 280 differentially expressed genes in at least one dataset. Next, a heatmap of the expression correlation of these differential genes across TCGA, GSE55696, and GSE79973 was generated (**Figure 2C**). Finally, these genes underwent univariate Cox analysis in TCGA, five additional GEO validation datasets were used, and a forest plot was constructed (**Figure 2D**). Twelve prognostic genes were ultimately selected, which showed prognostic significance in at least three datasets.

### 3.2 Functional characterization and molecular subtyping

**Figure 3:**
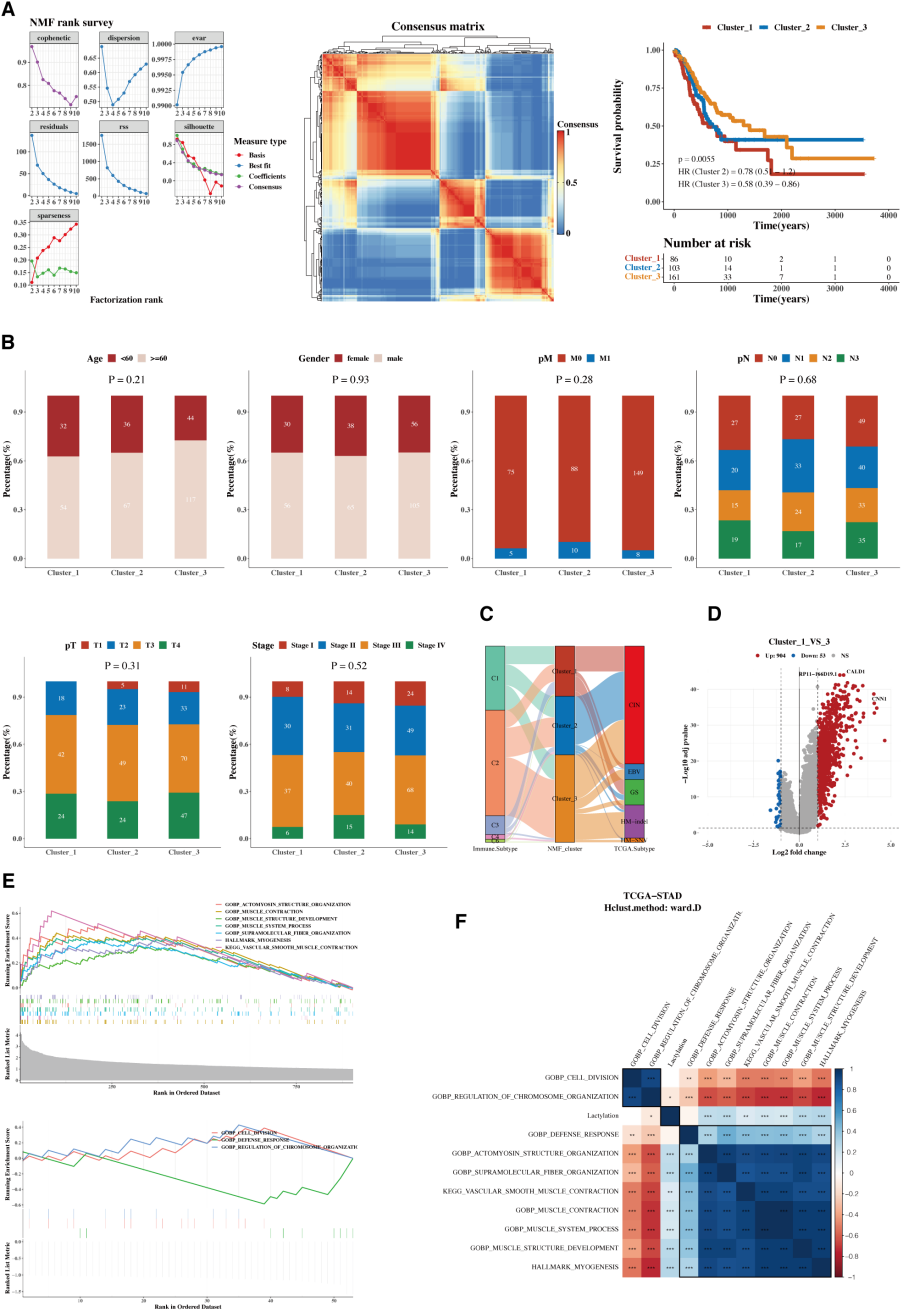
Functional characterization and molecular subtyping. **(A)** NMF clustering results, consistency heatmap, and survival analysis for 12 prognostic genes. Clusters C1, C2, and C3 represent gene clusters identified based on expression patterns in the cohort. The clustering was performed using hierarchical clustering, and the genes within each cluster exhibit distinct expression profiles. **(B)** Bar charts of clinical indicators such as age, gender, stage, and pathological grading in NMF subgroups. **(C)** Sankey diagram showing the composition of immune subtyping from TCGA and NMF grouping. **(D)** Volcano plot of gene differences between groups C1 and C3. **(E)** GSEA plots for upregulated and downregulated genes. **(F)** Heatmap of pathway enrichments correlated with ssGSEA scores of 12 lactylation genes.

Using the Non-negative Matrix Factorization (NMF) algorithm, the 12 prognostic genes were consistently clustered. Clustering results indicated that dividing into three groups was most appropriate. A consistency clustering heatmap and survival analysis results for the three groups are shown, with significant survival differences between groups C1 and C3, where C1 is associated with a poorer prognosis (**Figure 3A**). Further analysis was conducted to compare the composition of clinical indicators such as age, gender, stage, and pathological grading among the three groups, revealing differences that were not statistically significant (**Figure 3B**). A comparison of immune subtypes from TCGA with NMF grouping was also performed (**Figure 3C**). Due to the significant survival differences between C1 and C3, a differential gene analysis was conducted (**Figure 3D**). Gene enrichment analysis was performed separately for upregulated and downregulated genes, focusing on the functions associated with C1 and C3 (**Figure 3E**). The pathways enriched from these genes were calculated for their ssGSEA scores related to the 12 lactylation genes, and a correlation heatmap analysis was performed (**Figure 3F**).

### 3.3 Functional characterization—single cell and spatial transcriptomics

**Figure 4:**
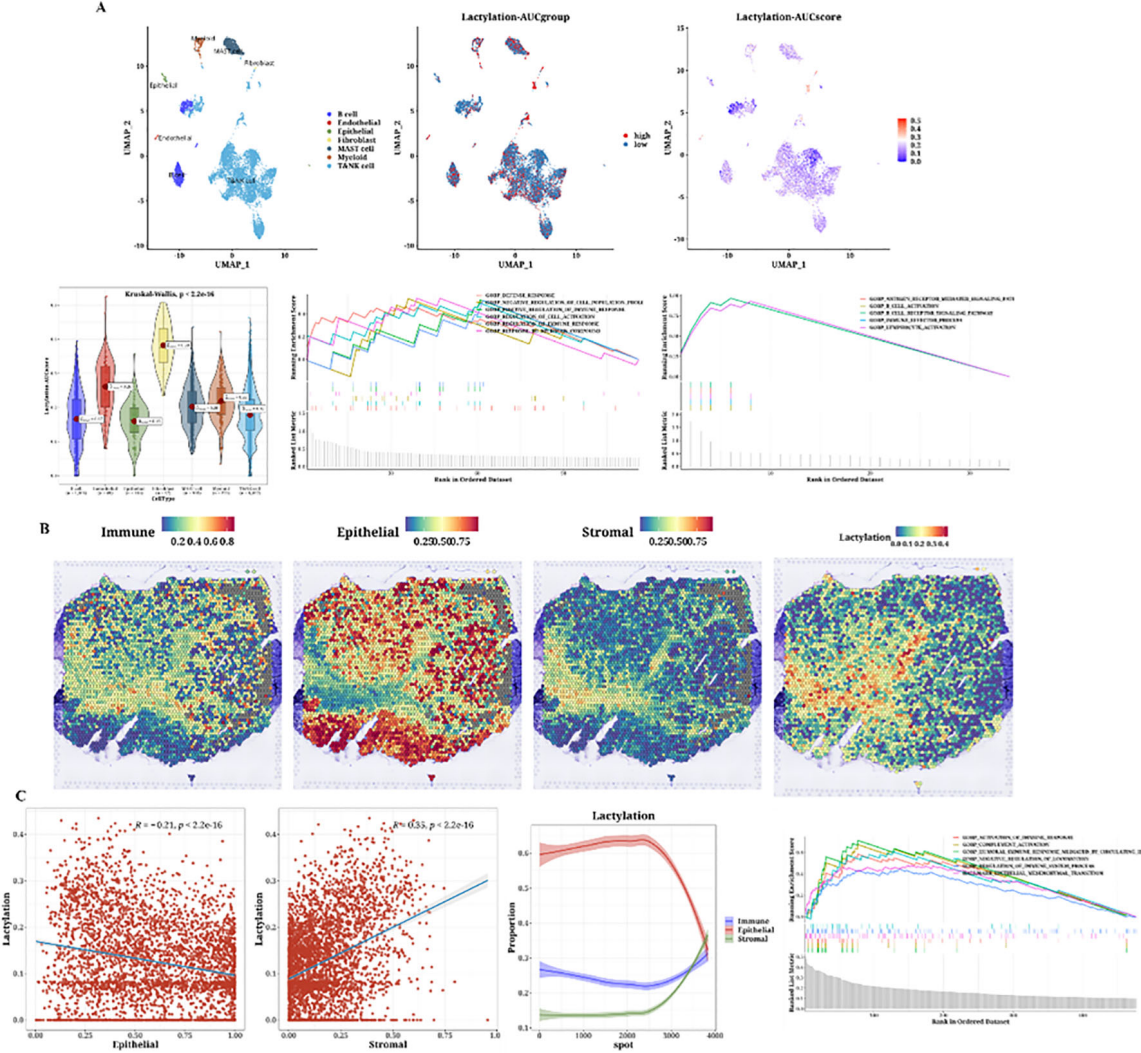
Single-cell analysis of lactylation-related gene expression and its association with immune, epithelial, and stromal cell populations in the tumor microenvironment. **(A)** Single-cell analysis showing lactylation scores across various cell types, including immune cells, fibroblasts (considered as stromal cells), and epithelial cells, lactylation grouping, UMAP plots of lactylation scores, violin plots of lactylation analysis, and GSEA plots for functional enrichment in high and low lactylation groups. The gradient from blue to cyan reflects a continuum of lactylation levels, indicating transitional states between low (blue) and high (cyan) lactylation scores among the cell populations. **(B)** H&E staining images of immune, epithelial, and stromal cells and lactylation scores in spatial transcriptomics data. **(C)** Correlation plots of epithelial (Pearson’s r = -0.21, p < 2.2e-16) and stromal scores (Pearson’s r = 0.53, p < 2.2e-16) with lactylation, curve plots of epithelial, immune, and stromal scores arranged by ascending lactylation, and GSEA plots for high lactylation functional enrichment.

The cell classification results from single-cell data are displayed, using the 12 prognostic genes with the AUCell package to calculate lactylation scores in each cell. Cells classified as stromal, including fibroblasts and other supportive tissue types within the tumor microenvironment, exhibited higher lactylation scores compared to epithelial cells. The function AUCell_exploreThresholds within the AUCell package was used to determine thresholds and divide cells into two groups. Differential gene and enrichment analyses were conducted to explore functional differences between these groups (**Figure 4A**). We present the analysis results of a spatial transcriptomics sample, showing the distribution differences of immune, epithelial, and stromal cells. Lactylation scores in the spatial samples were also calculated, revealing high lactylation areas, primarily in the stromal regions, consistent with the single-cell results (**Figure 4B**). The results indicated a negative correlation between epithelial cells and lactylation scores, while immune and stromal cells showed positive correlations. Subsequently, functional enrichment analysis for the high lactylation group was conducted (**Figure 4C**).

### 3.4 Development of a prognostic model based on differential genes

**Figure 5:**
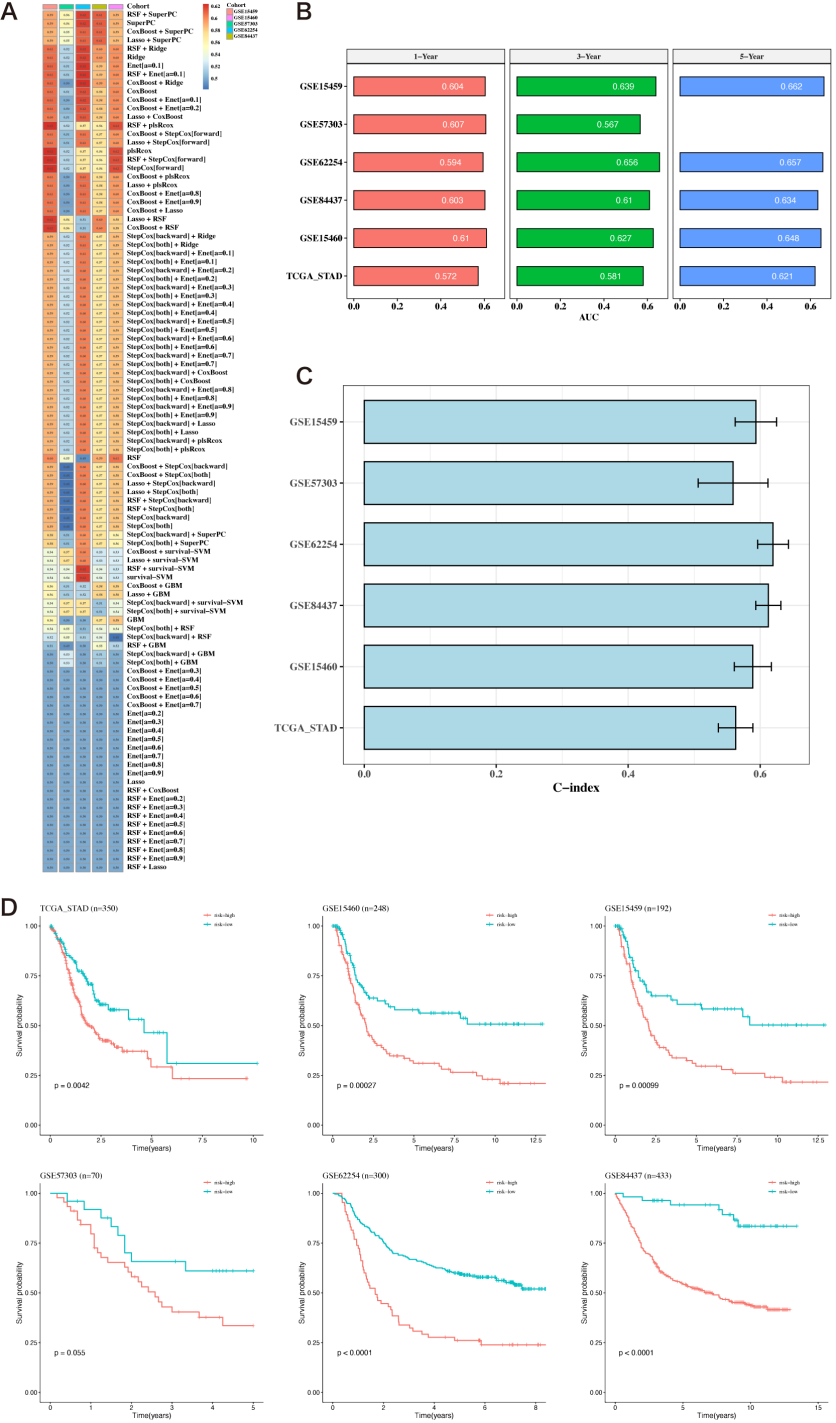
Construction of prognostic models based on differential genes. **(A)** Heatmap of C-indexes for 101 algorithms and five validation datasets. **(B)** AUC values for 1, 3, and 5 years across six datasets. **(C)** Bar chart of the optimal model’s C-index across various datasets. The error bars represent the standard error of the C-index values across these datasets. **(D)** Survival analysis results for six datasets.

Using the 12 prognostic genes, 101 algorithms were used to build models; the training set was TCGA, and the testing sets were five GEO datasets. The best model, determined by the average C-index across the five testing sets, was identified as RSF+SuperPC (**Figure 5A**). The AUC values for 1, 3, and 5 years were computed using the six datasets (**Figure 5B**). Bar charts displaying the C-index of the optimal model across different datasets are shown (**Figure 5C**). The survival analysis results from the six datasets indicated that the high-risk group had a poorer prognosis (**Figure 5D**).

### 3.5 Comparison of prognostic models

**Figure 6:**
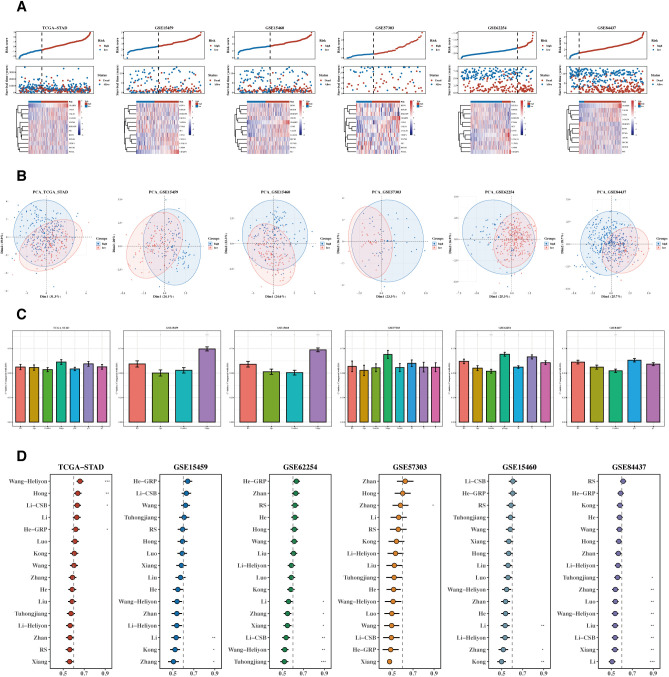
Comparison of prognostic models. **(A, B)** Risk plots and PCA diagrams for six datasets. Heatmap showing the expression of the 12 prognostic genes across patient samples in the TCGA-STAD cohort. Rows represent genes, and columns represent patient samples. The colors indicate the gene expression levels, with darker colors representing higher expression. The heatmap illustrates the association between gene expression and risk scores. **(C)** Bar chart of C-indexes comparing risk scores with other clinical indicators. **(D)** C-index chart comparing our prognostic model with 15 other recent models across six datasets.

Risk and PCA plots for the six datasets are presented (**Figures 6A, B**). Subsequently, risk scores were compared with other clinical indicators, and the risk score’s C-index was found to be superior to most clinical indicators (**Figure 6C**). We then collected 15 prognostic models published in the last 1-2 years and compared their C-index. While our prognostic model did not perform the best in the TCGA cohort, it generally outperformed most other models in the remaining five testing datasets (**Figure 6D**).

### 3.6 Development of the nomogram model

**Figure 7:**
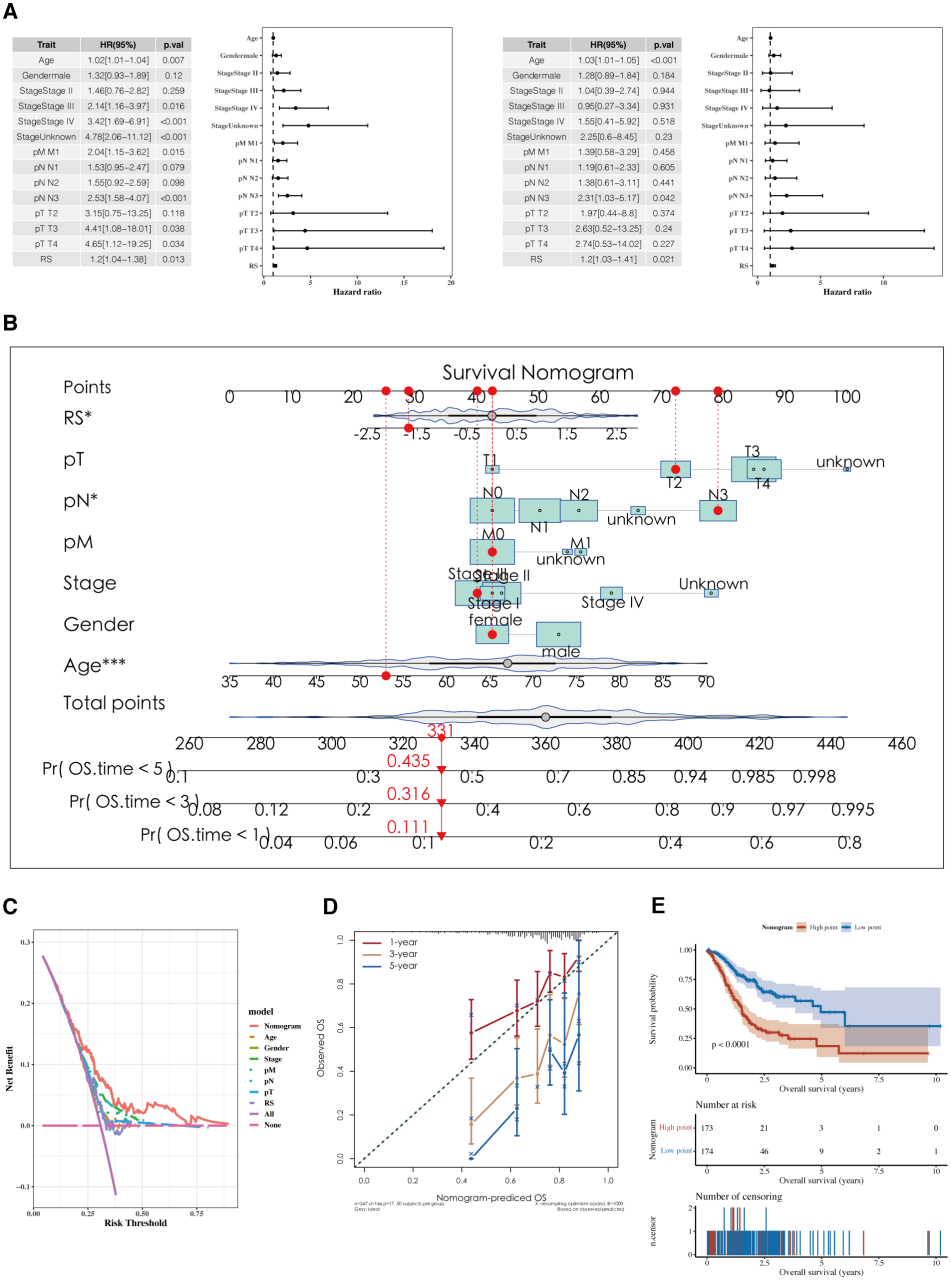
Development of a nomogram model. **(A)** Forest plots of univariate and multivariate analysis results for risk scores and clinical indicators. **(B)** Nomogram integrating risk scores with clinical indicators. **(C, D)** DCA plots and calibration curves for 1, 3, and 5 years. **(E)** Survival analysis results using Nomogram scoring.

Results of the analyses, both univariate and multivariate, of risk scores and clinical indicators are shown (**Figure 7A**) with the corresponding forest plots. A nomogram incorporating risk scores and clinical indicators is displayed (**Figure 7B**). Decision curve analysis (DCA) reveals that the outcomes from the nomogram and risk scores demonstrate superior performance compared to other clinical indicators (**Figure 7C**). Calibration curves for 1, 3, and 5 years are presented (**Figure 7D**). Survival analysis using the nomogram scores found that higher scores are associated with poorer prognosis (**Figure 7E**).

### 3.7 Tumor immune infiltration and TMB analysis

**Figure 8:**
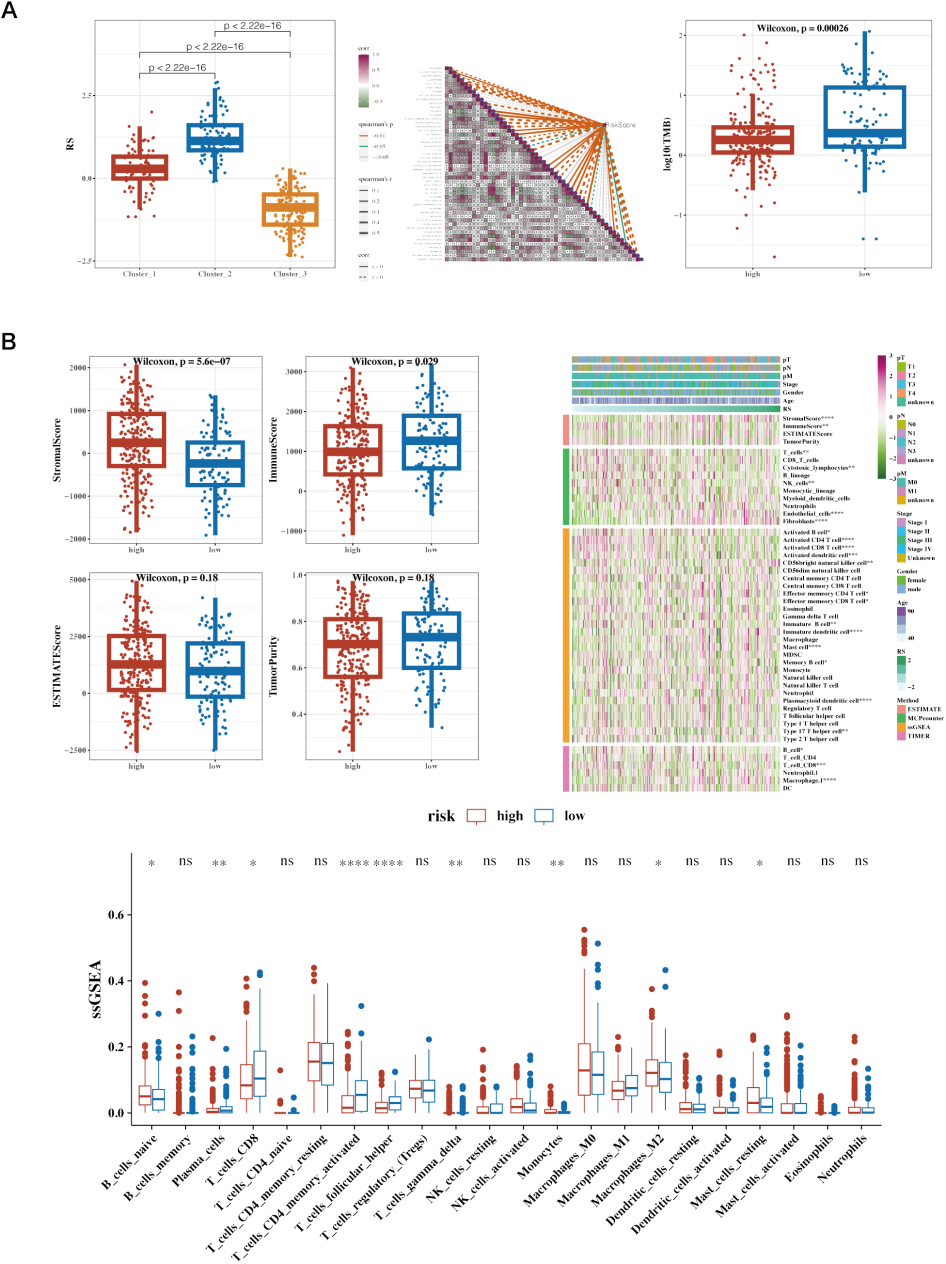
Immune infiltration and tumor mutation burden (TMB) analysis across different risk groups in cancer patients. **(A)** Boxplot of Risk Scores (RS) across Clusters, Correlation Heatmap of Gene Expression and Boxplot of Log10(TMB) in High vs. Low-Risk Groups. **(B)** Boxplots of Various Immune Scores in High vs. Low-Risk Groups. StromalScore: Measures the presence of stromal cells in tumor tissue. ImmuneScore: Quantifies the infiltration of immune cells in the tumor microenvironment. ESTIMATEScore: Represents the combined presence of stromal and immune cells. TumorPurity: Estimates the proportion of tumor cells in the sample. Heatmap of Immune Cell Infiltration and ssGSEA Results of Immune Cell Populations. Heatmap showing immune cell infiltration levels across patient samples. Rows represent different immune cell types, and columns represent patient samples. The color intensity reflects the level of immune cell infiltration, with darker colors indicating higher infiltration. This heatmap helps to visualize the relationship between immune infiltration and risk stratification.

Risk values for the three NMF classified groups are displayed, showing significant differences (**Figure 8A**). A correlation analysis was conducted between risk scores and the ‘50 hallmark gene sets’ from the Molecular Signatures Database (MSigDB), which represent distinct biological states and processes, commonly used to assess pathway activity in cancer research (**Figure 8A**). Tumor Mutational Burden (TMB) was calculated using mutation data, revealing significant differences among risk groups (**Figure 8A**). The differences between the two groups’ stromal, immunologic, and ESTIMATE scores are shown. The immune cell infiltration variations between the groups were depicted using the CIBERSORT algorithm. Further estimations of immune infiltration levels were made using other algorithms such as MCP-counter and TIMER, correlating them with risk scores, and displayed using a heatmap (**Figure 8B**).

### 3.8 Immunotherapy analysis and drug sensitivity analysis

**Figure 9:**
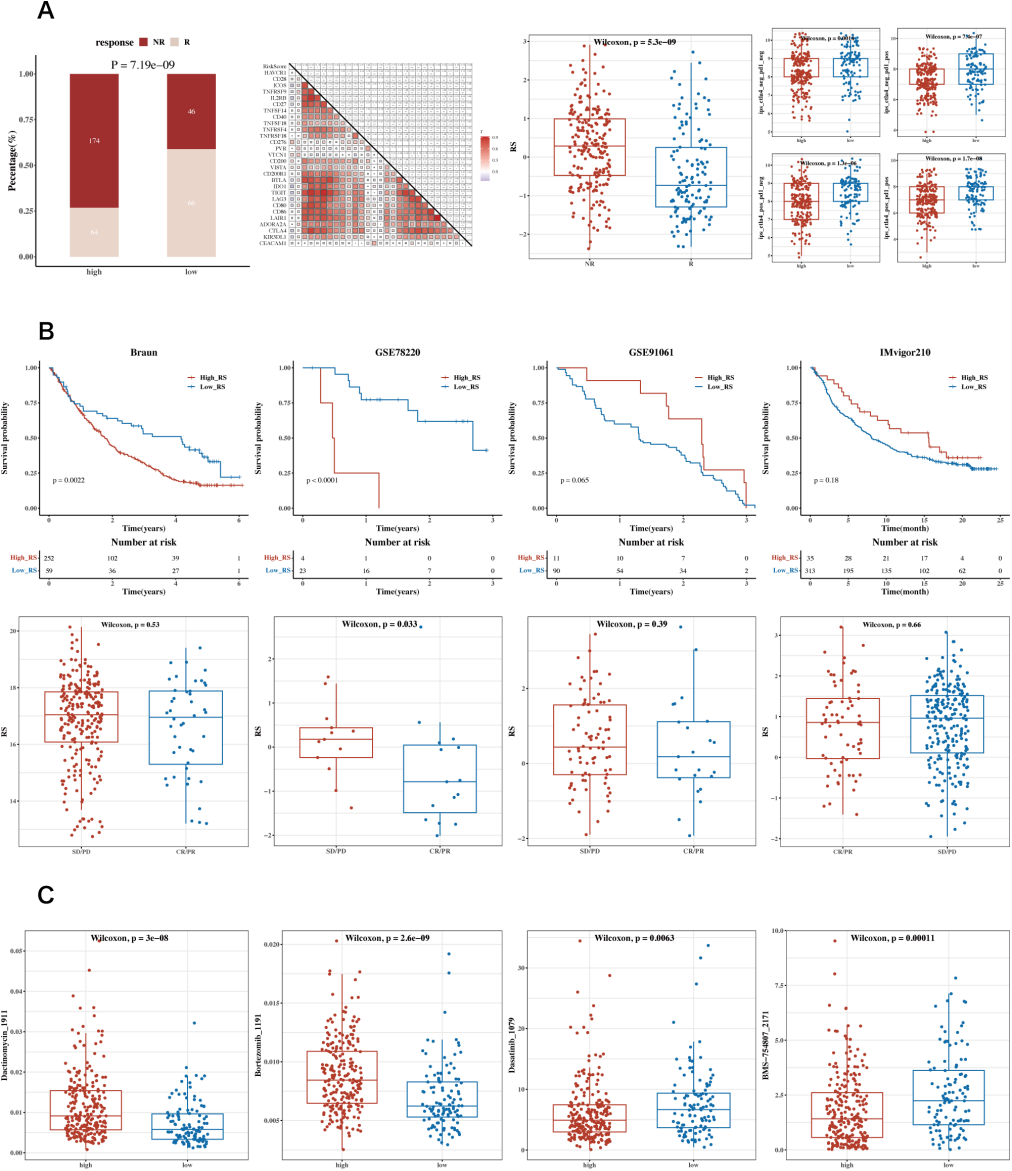
Analysis of immune therapy and drug sensitivity. **(A)** Heatmap of correlations between risk scores and immune checkpoint genes, bar charts for TIDE composition, box plots of TIDE risk values, and IPS box plots. **(B)** Survival analysis results and risk scores for immune response groups in GSE91061 (lung adenocarcinoma), GSE78220 (lung adenocarcinoma), IMvigor210 (urothelial carcinoma, UC), and Braun (renal cell carcinoma, RCC) datasets. **(C)** Box plots showing differential sensitivity to Bortezomib_1191, Dactinomycin_1911, Dasatinib_1079, and BMS-754807_2171 between high and low-risk groups.

Correlation analysis was conducted between immune scores and commonly used immune checkpoint genes, showing mostly negative correlations. Using the TCGA dataset, the TIDE algorithm was utilized to predict immune response scenarios. The results showed notable variations in the response compositions of the two risk groups, with the non-responsive group exhibiting higher risk scores. Results incorporating the Immune Phenotype Score (IPS) indicated that the low-risk group had higher IPS scores (**Figure 9A**). Survival analysis results from datasets including GSE91061 (lung adenocarcinoma), GSE78220 (lung adenocarcinoma), IMvigor210 (urothelial carcinoma, UC), and Braun (renal cell carcinoma, RCC) are displayed along with the risk scores for the two immune response groups (**Figure 9B**). Drug sensitivity analysis showed that Bortezomib_1191 and Dactinomycin_1911 were sensitive in the low-risk group, while Dasatinib_1079 and BMS-754807_2171 were sensitive in the high-risk group (**Figure 9C**). The analysis of GEO datasets from lung adenocarcinoma and urothelial carcinoma revealed similar trends in immune cell infiltration and responsiveness associated with lactylation-related gene expressions, suggesting that these variations could have broader implications beyond STAD. These findings support the hypothesis that lactylation may play a universal role in modulating the tumor immune microenvironment across different cancer types.

### 3.9 Differential cell communication in single-cell high and low prognostic risk cells

**Figure 10:**
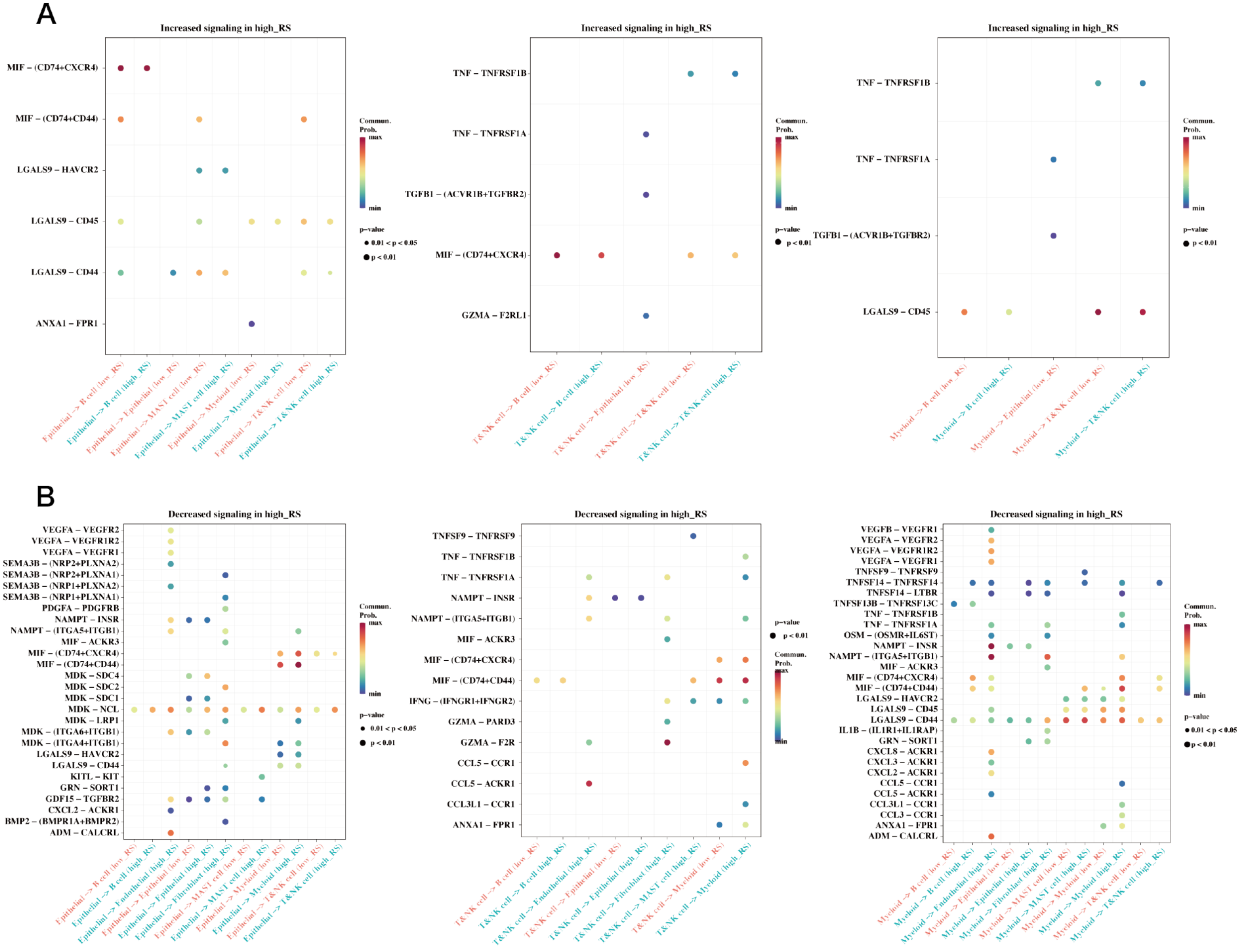
Differences in cell communication between high and low prognostic risk cells at the single cell level. **(A)** Bubble plot demonstrating enhanced cell communication differences between high-risk and low-risk groups in myeloid, epithelial, and T&NK cells. **(B)** A bubble plot illustrates the reduction in cell communication differences between high-risk and low-risk groups in T&NK, myeloid, and epithelial cells.

In the single-cell RNA sequencing dataset GSE184198, risk scores were calculated using the risk model for each cell, and the median value was used to group the cells. A Cellchat cell communication study was then conducted to compare the differences between the two groups (**Figure 10**). The differences in communication between the groups between epithelial, myeloid, and T & NK cells are displayed.

### 3.10 Expression of PTMA in tissues and cells

Based on our differential gene expression analysis, the PTMA gene was identified as significantly overexpressed in gastric cancer tissues compared to adjacent non-cancerous tissues. Among the 12 identified prognostic genes, most were part of the initial set of 304 lactylation-related and mitochondrial-related genes. However, PTMA, while not included in the initial sets of 332 lactylation-related genes or 170 mitochondrial-related genes, was identified as one of the 12 prognostic markers based on its significant association with mitochondrial dysfunction and its role in cancer progression, as determined through univariate Cox analysis. Subsequent RNA extraction from clinical samples confirmed the elevated expression of PTMA in tumor tissues, as demonstrated in **Figure 1A**. Furthermore, PTMA expression was determined in four gastric cancer cell lines (AGS, NCI-N87, BGC-823, and MKN45) and the normal gastric mucosa cell line GES-1. The findings were consistent with the trend observed in human tissues, indicating higher PTMA expression in gastric cancer cells (**Figure 1B**). These results suggest that the PTMA gene is highly expressed in gastric cancer.

### 3.11 Silencing PTMA inhibited the malignant behavior of gastric cancer cells

**Figure 11:**
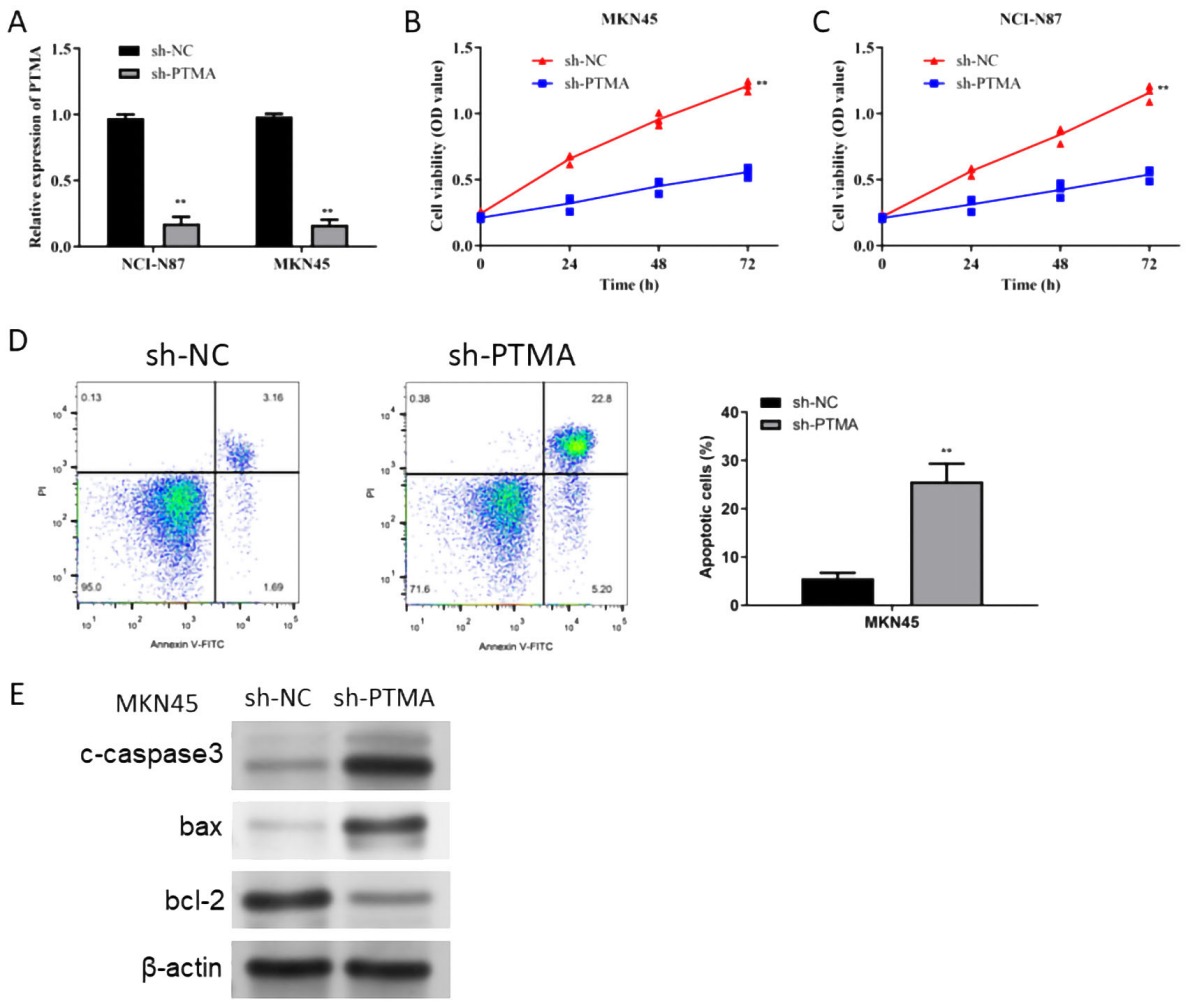
**(A)** RT-qPCR detected the knock-down efficiency of PTMA in NCI-N87 and MKN45 cell lines. **(B)** Cell viability of the MKN45 cell line before and after PTMA knockdown was detected by CCK8. **(C)** The cell viability of the NCI-N87 cell line before and after PTMA knockdown was detected by CCK8. **(D)** The apoptosis level of the MKN45 cell line before and after PTMA knockdown was detected by flow cytometry. **(E)** Western blot analysis assessed the expression of apoptosis-related proteins before and after PTMA knockdown in the MKN45 cell line. “**” denotes statistical significance (“**” *p* < 0.01). Sample sizes are indicated within the plots. Statistical comparisons were made using the Analysis of Variance (ANOVA).

The PTMA gene, one of the 12 identified prognostic markers, exhibited significant overexpression in gastric cancer tissues compared to normal tissues. Functional assays demonstrated that PTMA knockdown led to reduced proliferation, increased apoptosis, and decreased migration and invasion of gastric cancer cells, aligning with its predicted role in modulating tumor behavior as suggested by the bioinformatic analysis. To explore the role of the PTMA gene in STAD, we selected two cell lines, MKN45 and NCI-N87, and knocked down the PTMA gene (**Figure 11A**). As shown in **Figures 11B, C**, the proliferation ability of both gastric cancer cell lines was significantly reduced at all time points following PTMA knockdown. **Figure 11D** demonstrates that the apoptosis rate of tumor cells notably increased after PTMA knockdown. Furthermore, PTMA knockdown significantly raised the expression of the apoptosis-promoting proteins Bax and c-caspase3 while lowering the expression of the apoptosis-inhibiting protein Bcl-2, according to Western blot data (**Figure 11E**). This suggests that PTMA knockdown promotes apoptosis in gastric cancer cells.

**Figure 12:**
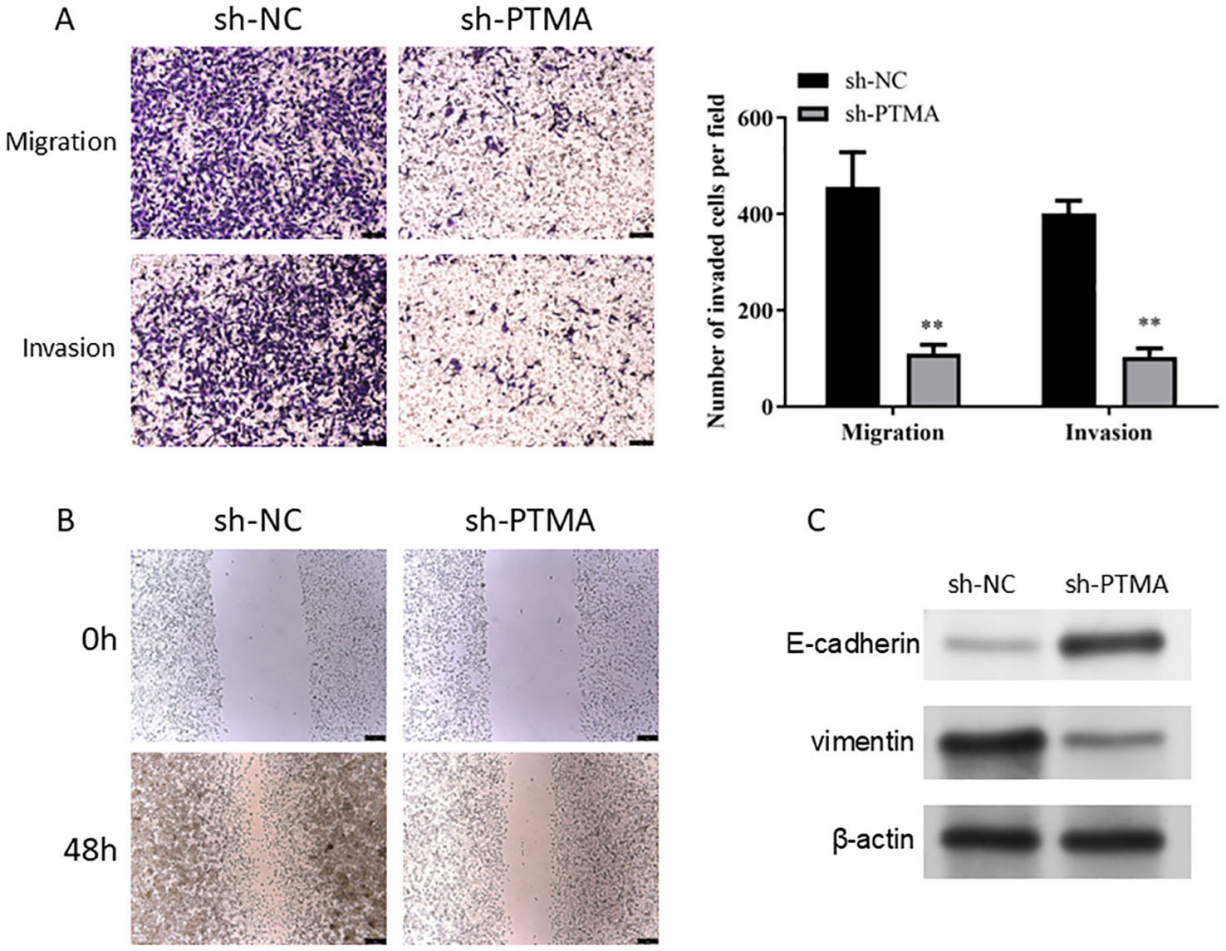
**(A)** A Transwell assay detected cell migration and invasion capability alterations before and after PTMA knockdown. **(B)** The capacity of cells to migrate was tested using the wound healing assay before and after PTMA knockout. **(C)** Western blot analysis of the changes in the expression levels of invasion and migration-related proteins before and after PTMA knockdown. “**” denotes statistical significance (“**” *p* < 0.01). Sample sizes are indicated within the plots. Statistical comparisons were made using the ANOVA.

In the Transwell invasion and migration assays (**Figure 12A**), MNK45 with PTMA knockdown exhibited significantly reduced invasion and migration abilities. Wound healing assays (**Figure 12B**) also showed a marked reduction in migration following PTMA knockdown. Western blot data shows PTMA knockdown expression with decreased Vimentin protein expression and increased E-cadherin protein expression (**Figure 12C**). These results strongly suggest that inhibiting PTMA expression negatively correlates with the malignant behavior of gastric cancer cells.

## 4 Discussion

The study presented here offers a comprehensive analysis of lactylation-related gene sets and mitochondrial-related genes in the context of gastric adenocarcinoma (STAD), leveraging large datasets like TCGA and various GEO datasets. A greater understanding of the tumor microenvironment and the cellular dynamics at work is made possible by integrating single-cell RNA sequencing and spatial transcriptomics, which is essential for improving our comprehension of STAD’s molecular pathogenesis and therapeutic responses. This discussion will highlight the findings, contrast them with existing research, and consider the implications of these results for future gastric cancer research and treatment strategies. Our findings indicate that lactylation-related genes significantly influence mitochondrial function and metabolic reprogramming in STAD. Specifically, lactylation can enhance glycolysis by modifying key glycolytic enzymes and histones, which promotes the expression of glycolytic genes and supports cancer cell proliferation. Additionally, by altering mitochondrial proteins and enzymes involved in oxidative phosphorylation (OXPHOS) and the TCA cycle, lactylation contributes to a metabolic shift favoring glycolysis. This metabolic reprogramming is a hallmark of cancer cells, allowing them to thrive in the hypoxic tumor microenvironment. These insights suggest that targeting lactylation and its related metabolic pathways could offer new therapeutic strategies for STAD by disrupting cancer cell metabolism and enhancing the efficacy of existing treatments.

Our study has highlighted several critical areas in the pathology of STAD. While our findings indicate significant correlations between lactylation-related gene expressions and mitochondrial-related genes, it is important to note that these results are exploratory. The lactylation-related gene sets offer a novel perspective on metabolic reprogramming in cancer cells and its potential impact on tumor behavior, which warrants further investigation to establish any causal relationships. First, the prognostic gene sets identified through differential expression analysis and their correlation with patient outcomes provide valuable insights into the biological underpinnings of STAD. The lactylation-related gene sets offer a novel perspective on metabolic reprogramming in cancer cells and its impact on tumor behavior. Second, single-cell and spatial transcriptomics have uncovered significant heterogeneity within the tumor microenvironment, especially in the distribution and role of stromal and immune cells, which are pivotal in modulating tumor progression and response to therapies. By extending our analysis to include lung adenocarcinoma and urothelial carcinoma, we demonstrated that the variations in lactylation-related gene expressions and their effects on immune response might be applicable across multiple cancer types. This not only reinforces our findings in STAD but also opens avenues for further research into the universal roles of lactylation in cancer biology. The experimental validation of PTMA underscores its potential as a key regulator in gastric adenocarcinoma. The results support its role in modulating both mitochondrial function and immune responses, which are critical aspects of cancer progression. These findings provide a foundation for considering PTMA as a therapeutic target, potentially enhancing the effectiveness of treatments that disrupt lactylation processes.

Recent studies have begun to explore the role of lactylation in cancers. For instance, Zhang et al. reported that lactylation of histone lysine residues could promote gene expression related to glycolysis in cancer cells, thereby facilitating cancer progression (29). Our findings align with this perspective, suggesting lactylation may play a similarly critical role in STAD. However, our research extends this by mapping specific lactylation-related genes that correlate with prognosis, which has not been extensively documented in previous studies. Our study further refines molecular subtyping in STAD by incorporating novel biomarkers from our analysis. This approach has been foundational in studies like The Cancer Genome Atlas Research Network’s 2014 publication, which classified gastric cancer into four molecular subtypes. While this classification has significantly advanced the field, our study provides additional layers of molecular characterization, particularly highlighting the importance of metabolic reprogramming and mitochondrial dysfunction. Our prognostic models, built on differential gene expression and validated across multiple datasets, have shown superior performance compared to many existing models. For instance, Cristescu et al. developed a prognostic model that utilized gene expression data but did not incorporate the latest single-cell and spatial profiling technologies, which may account for the enhanced accuracy of our models (30).

The immune landscape of STAD, particularly as related to immunotherapy, has been a focus of recent research. A study by Thorsson et al. highlighted the variability of the immune environment across cancers and its implications for immunotherapy (31). In addition to our findings on lactylation-related pathways in STAD, it is important to consider broader therapeutic and diagnostic implications for gastric cancer treatment. For example, understanding the incidence and outcomes of secondary infections in septic cancer patients is crucial for managing complications associated with STAD treatment (32). Incorporating this knowledge could lead to more effective and holistic patient management strategies. Furthermore, investigating traditional herbal medicines as adjunctive therapies has shown potential in enhancing treatment outcomes in colorectal cancer, and similar strategies might be applicable to gastric cancer to improve therapeutic efficacy and patient outcomes (33). Recent advances in cancer nanotechnology, such as the development of dendrimeric nanosystems and mesoporous silica/organosilica nanoparticles, have shown great promise in overcoming drug resistance and improving cancer immunotherapy (34, 35). These innovative technologies could be applied to target lactylation-related pathways, potentially enhancing the therapeutic effectiveness for STAD. Additionally, emerging diagnostic techniques like single-exosome profiling have identified specific exosome subpopulations as early diagnostic biomarkers and therapeutic targets in colorectal cancer (36). Utilizing similar approaches in STAD could facilitate earlier detection and more targeted treatment strategies.

Moreover, the therapeutic potential of natural products derived from various microorganisms for treating cancers, such as cervical cancer, underscores the importance of exploring diverse therapeutic avenues (37). These natural products could be repurposed for STAD treatment, offering new, less toxic options for patients. Finally, targeting specific proteins, such as HJURP, which play key roles in cancer progression across multiple types of cancer, could provide new insights and pathways for developing targeted therapies in STAD (38). These broader perspectives highlight the need for future research to adopt a multifaceted approach that integrates molecular findings with advanced therapeutic strategies, ultimately enhancing the effectiveness of cancer treatments and improving patient outcomes.

Our analysis complements this by showing how lactylation influences the immune microenvironment in STAD, providing a potential link between metabolic states and immune responsiveness. This could lead to more tailored immunotherapeutic strategies that consider the tumor’s molecular and immunological profiles.

The findings from our study on STAD emphasize the potential for novel research directions, particularly in targeting metabolic pathways and enhancing immunotherapy efficacy. Given the significant role that lactylation and mitochondrial functions play in STAD, future therapeutic strategies could involve the development of inhibitors that specifically disrupt lactyl-CoA production or the lactylation process itself, aiming to impair the tumor’s ability to thrive under metabolic stress. Moreover, understanding the interaction between lactylation and the immune microenvironment offers opportunities to enhance the efficacy of immunotherapy. Modifying lactylation levels may increase the visibility of cancer cells to immune cells, potentially making immunotherapies more effective. While our study primarily focuses on the role of lactylation-related genes in STAD, we acknowledge the potential interactions between lactylation and other post-translational modifications (PTMs), such as acetylation and phosphorylation. These interactions could have significant implications for mitochondrial function and tumor metabolism. Although a comprehensive investigation into these crosstalk mechanisms is beyond the scope of the present study, we propose this as an important direction for future research to further elucidate the regulatory networks involved in cancer metabolism. Additionally, our study’s detailed molecular and cellular characterization supports the advancement of precision medicine approaches. By identifying specific molecular drivers and cellular interactions within individual tumors, treatments can be more effectively tailored to the unique characteristics of each patient’s cancer, offering a pathway to more personalized and effective treatment strategies for STAD. While the prognostic model developed in this study demonstrates strong predictive power, its translation into clinical practice presents several challenges. First, the requirement for advanced genomic sequencing and specialized bioinformatics analyses may not be feasible in all clinical settings, potentially limiting its immediate application. Additionally, variability in gene expression across different patient populations poses a challenge to the model’s generalizability. To address these limitations, further validation in diverse patient cohorts and the development of more accessible testing methodologies are necessary. Furthermore, standardization of protocols and compliance with regulatory requirements will be crucial for the successful integration of this model into clinical practice. Future efforts will focus on overcoming these barriers to facilitate the clinical adoption of this prognostic tool, with the aim of enhancing personalized treatment strategies for patients with gastric adenocarcinoma.

This study enriches the current understanding of gastric adenocarcinoma through an intricate gene expression analysis, especially focusing on novel areas like lactylation. The application of cutting-edge technologies has uncovered layers of complexity within the tumor microenvironment previously unexplored in such depth. By contrasting these findings with existing literature, it is evident that this research not only corroborates many known aspects of gastric cancer but also provides new avenues for therapeutic intervention and prognostic evaluation. As we move forward, it will be essential to integrate these findings into clinical trials and therapeutic development to truly transform patient care in gastric adenocarcinoma.

## Data Availability

The original contributions presented in the study are included in the article/supplementary material. Further inquiries can be directed to the corresponding authors.
